# Association of healthy lifestyles on the risk of hypertension, type 2 diabetes mellitus, and their comorbidity among subjects with dyslipidemia

**DOI:** 10.3389/fnut.2022.1006379

**Published:** 2022-09-26

**Authors:** Peng Hu, Murui Zheng, Xueru Duan, Huanning Zhou, Jun Huang, Lixian Lao, Yue Zhao, Yi Li, Meng Xue, Wenjing Zhao, Hai Deng, Xudong Liu

**Affiliations:** ^1^School of Public Health, Guangdong Pharmaceutical University, Guangzhou, China; ^2^Department of Epidemiology, School of Public Health, Sun Yat-sen University, Guangzhou, China; ^3^Department of Community Health, Guangzhou Center for Disease Control and Prevention, Guangzhou, China; ^4^Department of Chronic Disease, Guangzhou Yuexiu District Center for Disease Control and Prevention, Guangzhou, China; ^5^Department of Geriatrics, Institute of Geriatrics, Guangdong Provincial People's Hospital, Guangdong Academy of Medical Science, Guangzhou, China; ^6^Department of Cardiology, Guangdong Cardiovascular Institute, Guangdong Provincial People's Hospital, Guangdong Academy of Medical Science, Guangzhou, China; ^7^School of Public Health and Emergency Management, Southern University of Science and Technology, Shenzhen, China

**Keywords:** hypertension, diabetes, comorbidity, subjects with dyslipidemia, lifestyle

## Abstract

**Background:**

Adherence to a healthy lifestyle could reduce the risk of hypertension and diabetes in general populations; however, whether the associations exist in subjects with dyslipidemia remains unclear. This study aimed to investigate the integrated effect of lifestyle factors on the risk of hypertension, type 2 diabetes mellitus (T2DM), and their comorbidity among subjects with dyslipidemia.

**Methods:**

In total of 9,339 subjects with dyslipidemia were recruited from the baseline survey of the Guangzhou Heart Study. A questionnaire survey and medical examination were performed. The healthy lifestyle score (HLS) was derived from five factors: smoking, alcohol drinking, diet, body mass index, and leisure-time physical activity. Odds ratios (ORs) with 95% confidence interval (95% CI) were calculated by using the logistic regression model and the multinomial logistic regression after adjusting for confounders.

**Results:**

The prevalence of hypertension, T2DM, and their comorbidity was 47.65, 16.02, and 10.10%, respectively. Subjects with a higher HLS were associated with a lower risk of hypertension, T2DM, and their comorbidity. In comparison to the subjects with 0–2 HLS, the adjusted ORs for subjects with five HLS was 0.48 (95% CI: 0.40–0.57) and 0.67 (95% CI: 0.54–0.84) for hypertension and T2DM. Compared with subjects with 0-2 HLS and neither hypertension nor T2DM, those with five HLS had a lower risk of suffering from only one disease (OR: 0.48, 95% CI: 0.40–0.57) and their comorbidity (OR: 0.35, 95% CI: 0.26–0.47).

**Conclusions:**

The results suggest that the more kinds of healthy lifestyle, the lower the risk of hypertension, T2DM, and their comorbidity among subjects with dyslipidemia. Preventive strategies incorporating lifestyle factors may provide a more feasible approach for the prevention of main chronic diseases.

## Introduction

Previous studies have shown that dyslipidemia, hypertension, and type 2 diabetes mellitus (T2DM) are three key risk factors for cardiovascular diseases, and they often occur alone or synergistically as main chronic non-communicable diseases (1–3). They have become a major public health challenge, especially in developing countries (4, 5). According to the 2017 Global Burden of Disease Study, high systolic blood pressure and high fasting plasma glucose have become the leading risks of death, and the global burden of dyslipidemia has increased with socio-economic development (6). In China, the prevalence of dyslipidemia has constantly increased in recent years (7, 8). Previous studies have found that compared with those with normal blood lipid levels, patients with dyslipidemia were at higher risk for developing hypertension and diabetes, and even have a higher prevalence of coexisting risk factors (3, 9, 10). Given the commonality in lifestyle factors among hypertension, diabetes, and dyslipidemia, it is of significance to identify effective strategies to prevent or delay the development of hypertension and T2DM in subjects with dyslipidemia.

Based on published studies, efforts to reduce the risk of hypertension and T2DM, such as adherence to a healthy lifestyle, have been encouraged in the general population (11–14). For instance, previous studies conducted in different countries showed that four main healthy lifestyle factors including no smoking, no drinking, healthy physical activity, and healthy body mass index (BMI), were associated with a decreased risk of hypertension (11–13). A systematic review and meta-analysis including 14 studies and approximately one million subjects indicated that adherence to the healthiest lifestyles was associated with a 75% reduced risk of T2DM when compared with individuals with the least healthy lifestyles (14). However, in the subjects with dyslipidemia, whether adopting a healthy lifestyle will also have similar beneficial effects on hypertension and T2DM is still unclear.

Therefore, this present study aimed to examine the integrated effect of five major lifestyle factors, including smoking, alcohol drinking, diet, BMI, and leisure-time physical activity (LTPA), on the risk of hypertension, T2DM, and their comorbidity among subjects with dyslipidemia. The results of this study will provide evidence to prevent hypertension and T2DM by adopting lifestyle adjustment strategies that are easy to implement among subjects with dyslipidemia.

## Methods

### Setting and subjects

This study was based on the baseline survey of the Guangzhou Heart Study (GZHS), an ongoing prospective population-based cohort study in Guangzhou, China. The baseline survey of GZHS was successfully conducted between July 2015 and August 2017 using the multistage sampling method. Detailed information about GZHS can be seen in our previous reports (15–19). Briefly, a total of 12,013 permanent residents aged 35 years or above were recruited and accomplished the GZHS baseline survey. Those who had mental or cognitive disorders, had mobility difficulties, had any history of cancer, were pregnant or lactating women, and were non-Guangzhou permanent residents were excluded when recruiting subjects. In this study, among 12,013 subjects, we excluded subjects with missing data on blood pressure or diabetes-related information (*n* = 5) and subjects without dyslipidemia (*n* = 2,669). Ultimately, a total of 9,339 subjects with dyslipidemia were included for further analysis. The flow chart of selecting subjects was shown in Supplementary Figure S1.

This study was approved by the Ethical Review Committee for Biomedical Research, School of Public Health, Sun Yat-sen University. The study was performed in line with the Declaration of Helsinki and all subjects provided informed consent.

### Ascertainment of hypertension, T2DM, and dyslipidemia

All subjects were invited to undergo a free medical examination. Subjects were instructed to rest for 10 min in a quiet room before blood pressure measurement. Systolic blood pressure and diastolic blood pressure were measured three times by trained medical workers, and then the mean of the three measurements was calculated. Hypertension was defined as systolic blood pressure ≥ 140 mmHg or diastolic blood pressure ≤ 90 mmHg or having self-reported physician-diagnosed hypertension (19). A fasting blood sample from each subject was collected, and serum concentrations of fasting blood glucose, triglyceride, low-density lipoprotein cholesterol, and cholesterol were detected. Subjects whose fasting blood glucose was ≥ 7.0 mmol/L or who had self-reported physician-diagnosed diabetes (excluding gestational diabetes mellitus, type 1 diabetes mellitus, or other types of diabetes) were defined as having T2DM (20). Subjects who self-reported physician-diagnosed dyslipidemia or with serum cholesterol of ≥5.2 mmol/L, low-density lipoprotein cholesterol (LDL-C) of ≥ 3.4 mmol/L, high-density lipoprotein cholesterol (HDL-C) of < 1.0 mmol/L or triglyceride of ≥ 1.7 mmol/L was considered as having dyslipidemia (21). Uniform inclusion and exclusion criteria mentioned above were used for the three disease populations.

### Assessment of lifestyle factors

Structured questionnaires conducted with a face-to-face approach were used to collect information on social demographics and lifestyle factors including cigarette smoking and alcohol drinking. For smoking, subjects who have never smoked or smoked < 100 cigarettes in their lifetime were classified as non-smokers, and those who have currently smoked or smoked ≥100 cigarettes in their lifetime were classified as smokers. For alcohol drinking, subjects were asked to report their current drinking status: “frequent drinking”, “occasional drinking” and “never drinking or alcohol cessation”. Subjects who reported “frequent drinking” were considered as frequent drinkers, others as moderate drinkers.

Dietary consumption was collected using a 22-item food frequency questionnaire (FFQ) (17). The intake frequency of each food item (< once per month, 1–3 times per month, 1–3 times per week, 4–6 times per week, and ≥once per day) over the previous 12 months was collected from each subject. A total of 12 major food items in FFQ (cereals, legumes, vegetables, fruit, dairy, nuts, fish, poultry, red meat, fried foods, high-salt foods, and sugary beverages) were used to create a diet quality score based on the Chinese Dietary Guidelines (22). For fruit and vegetables, one point was assigned if they were consumed more than three times per week; for whole grains, legumes, nuts, dairy, poultry, and fish, one point was assigned separately if they were consumed at least once per week; for red meat, one point was assigned if it was consumed less than once per week; for high-salt foods, fried foods, and sugary beverages, one point was assigned separately if they were not consumed or consumed less than once per month. A point of 0 was assigned to each food item if the intake frequency did not meet the corresponding criteria aforementioned. Accordingly, the diet quality score of subjects ranged from 0 (lowest) to 12 (highest). A healthy diet was defined as a quality score was 7 points or more (the median value), otherwise an unhealthy diet.

The medical examination was performed on each participant; height and weight were measured using standard instruments. Body mass index (BMI) was calculated by dividing weight (kg) by height squared (m^2^); a healthy BMI was defined as BMI in the range of 18.5 to 23.9 kg/ m^2^ according to the Chinese standard, otherwise an unhealthy BMI (23).

Leisure-time physical activity (LTPA) was evaluated by a modified Global Physical Activity Questionnaire (24, 25). The total volume of LTPA for each subject was calculated as the sum of volumes of eight categories of most common LTPA including Tai Chi/Qigong, housework, stroll, bicycling, brisk walking/exercises/Yangko, ball games (basketball, table tennis, badminton, etc.), swimming, long-distance running/aerobics dancing. The volume of each LTPA was assessed by multiplying the frequency of activity by its duration and then by its intensity (quantified by the value of metabolic equivalent, MET). More detailed information on the assessment of physical activity was shown in our previous report (19). According to World Health Organization (WHO) guidelines on physical activity, to attain substantial health benefits, adults should perform at least 150–300 min of moderate-intensity aerobic physical activity, or at least 75–150 min of vigorous-intensity aerobic physical activity, or an equivalent combination of both throughout the week (26). Accordingly, conducting activity with at least 10 MET-hours/week is suggested as the minimum level of the recommended standard (26).

### Healthy lifestyle score establishment

The details of the healthy lifestyle score (HLS) are shown in Table 1. The HLS was established by using five modifiable lifestyle factors including smoking, alcohol drinking, diet, BMI, and LTPA. These factors were dichotomized as healthy or unhealthy, and each factor was assigned a score of 1 and 0 for healthy and unhealthy, respectively. The score for each lifestyle factor was defined as follows: smoking (1 = non-smoker, 0 = smoker), alcohol drinking (1 = moderate drinker, 0 = frequent drinker), diet (1 = healthy diet, 0 = unhealthy diet), BMI (1 = healthy BMI, 0 = unhealthy BMI), LTPA (1 = reach the minimum level of the WHO recommended standard, 0 = not reach the minimum level of the WHO recommended standard). The total score for HLS was calculated as the sum of the scores of five selected factors. Consequently, The HLS ranged from zero (least healthy) to five (healthiest) points.

**Table 1:** Description and scoring criteria of lifestyle factors.

**Lifestyle factor**	**Classification**	**Point**	**Description**
Smoking	Smoker	0	Current smoker or former smoker (≥100 cigarettes)
	Non-smoker	1	Never smoker or former smoker (< 100 cigarettes)
Alcohol drinking	Frequent	0	Frequent drinking
	Moderate	1	No drinking or occasionally drinking
Diet	Unhealthy	0	Diet quality score < 7
	Healthy	1	Diet quality score ≥ 7
Body mass index	Unhealthy	0	Overweight or obese (BMI < 18.5 kg/m^2^ or BMI ≥ 24 kg/m^2^)
	Healthy	1	Normal weight (18.5 ≤ BMI < 24 kg/m^2^)
Leisure-time physical activity	Unhealthy	0	Did not meet WHO guidelines on physical activity: less than 10.0 MET-h/week
	Healthy	1	Meet WHO guidelines on physical activity: 10.0 MET-h/week or above

BMI, body mass index; MET-h, metabolic equivalent values-hours; WHO, World Health Organization.

### Potential confounding factors

The structured questionnaires aforesaid were used to collect information on social demographics by using face-to-face interviews. The social demographics included age (years), sex (male, female), education (< high school, ≥high school), marital status (married, others), and retirement status (yes, no).

### Statistical analysis

The continuous variables with normal distribution were expressed using mean and standard deviation (SD), and the continuous variables with non-normal distribution were displayed using median and quartile range (IQR). The normality was examined by the Kolmogorov-Smirnov test. The distribution of categorical variables was represented as frequency and percentage. The distribution difference of baseline social demographics, lifestyle factors, and other covariables among different groups was evaluated by chi-square test for a categorical variable and *t*-test, one-way analysis of variance, Wilcoxon rank-sum test or Kruskal-Wallis rank sum-test for a continuous variable. The associations between HLS and its components were examined using the Spearman correlation coefficient (r_s_). Crude and adjusted odds ratios (ORs) and 95% confidence intervals (CIs) were measured by logistic regression models to demonstrate the individual and overall impact of lifestyle factors on the risk of hypertension and T2DM. Multinomial logistic regression was used to estimate ORs and CIs of suffering from either or both hypertension and T2DM compared with those of non-hypertension and non-T2DM. Stratified analysis was conducted by sex and retirement status. The multiplicative interaction of HLS with sex and retirement status was estimated respectively using the likelihood ratio test, with a comparison of the likelihood scores of the two models with and without the interaction terms.

A series of sensitivity analyses were conducted to examine the robustness of the results. We used every dyslipidemia indicator to define the dyslipidemia and repeated the analysis. Considering that different lifestyle components may have unequal effects on diseases, we created a weighted lifestyle score weighted by the multivariable-adjusted risk estimates (β coefficients) on hypertension and T2DM. The equation was: Weighted score = (β_×1_ factor_1_ + β_2_ × factor_3_ + β_3_ × factor_4_ + β_4_ × factor_4_ + β_5_ × factor _5_) × (5/sum of the β coefficients) (27). We further grouped all subjects into three categories based on the tertile cut-off points of weighted score. As the volume of LTPA of most subjects met the WHO recommended standard (85.43%), we conducted an analysis by using the median in place of the WHO-recommended cut-off value of LTPA in generating HLS. To verify the effect of different BMI cut-off values on our results, we further used 25 kg/m^2^, suggested as the cut-off point of overweight by WHO, as the cut-off value for healthy and unhealthy BMI in generating HLS. To examine whether the HLS was appropriate for the risk assessment (28), we replaced BMI with waist circumference as a component of HLS. Repeated analyses were also performed by excluding subjects with a BMI of less than 18.5 kg/m^2^ to rule out unknown bias brought by underweight (*n* = 355), by excluding subjects aged 75 years or above to rule out the effects of age-related factors (*n* = 953), and with additional adjustment for the number of dyslipidemia indicators. All analyses were conducted with R 4.0.1 (R Development Core Team, Vienna, Austria); the tests were two-tailed, and a *P*-value of less than 0.05 was considered statistically significant.

## Results

Of the total of 9,339 subjects with dyslipidemia, 4,889 subjects (52.35%) were divided into the non-hypertension group and 4,450 (47.65%) into the hypertension group; 7,843 (83.98%) subjects were classified into the non-T2DM group and 1,496 (16.02%) into the T2DM group; 4,336 (46.43%) subjects suffered neither hypertension nor T2DM and 943 (10.10%) suffered both hypertension and T2DM.

The mean age and BMI in the hypertension group and T2DM group were larger than those in the non-hypertension and non- T2DM groups respectively (Table 2). In comparison to the non-hypertension subjects, subjects with hypertension were more likely to be male and not married, to have a lower level of education, to be retirees, to smoke and drink alcohol, to have an unhealthy diet, or have an unhealthy BMI (all *P* < 0.05). More subjects in the diabetes group than in the non-T2DM group were male, married, retirees, and had an unhealthy BMI (all *P* < 0.05).

**Table 2:** Baseline characteristic of the subjects with dyslipidemia.

**Characteristic**	**Total (*N =* 9,339)**	**Non-hypertension** **(*N =* 4,889)**	**Hypertension (*N =* 4,450)**	***P***	**Non-T2DM (*N =* 7,843)**	**T2DM** **(*N =* 1,496)**	***P***
Age, years, mean (S.D.)	59.49 (11.44)	55.33 (10.50)	64.07 (10.65)	< 0.001*	58.78 (11.38)	63.22 (11.00)	< 0.001*
BMI, kg/m^2^, mean (S.D.)	24.21 (3.54)	23.41 (3.29)	25.08 (3.59)	< 0.001*	24.06 (3.48)	24.99 (3.72)	< 0.001*
LTPA, MET-h, median (Interquartile)	34.65 (41.10)	34.65 (42.07)	34.65 (40.25)	0.541^†^	34.65 (41.60)	35.00 (39.23)	0.928^†^
Diet quality score, mean (S.D.)	7.40 (1.80)	7.460 (1.80)	7.339 (1.81)	0.001*	7.385 (1.80)	7.495 (1.84)	0.033*
**Sex**, ***N*** **(%)**				< 0.001^‡^			0.041^‡^
Male	3,228 (34.56)	1,555 (31.81)	1,673 (37.60)		2,676 (34.12)	552 (36.90)	
Female	6,111 (65.44)	3,334 (68.19)	2,777 (62.40)		5,167 (65.88)	944 (63.10)	
**Education**, ***N*** **(%)**				< 0.001^‡^			0.180^‡^
< high school	5,809 (62.20)	2,744 (56.13)	3,065 (68.88)		4,902 (62.50)	907 (60.63)	
≥high school	3,530 (37.80)	2,145 (43.87)	1,385 (31.12)		2,941 (37.50)	589 (39.37)	
Material status, *N* (%)				< 0.001^‡^			0.003^‡^
Married	7,884 (84.42)	4,271 (87.36)	3,613 (81.19)		6,660 (84.92)	1,224 (81.82)	
Others	1,455 (15.58)	618 (12.64)	837 (18.81)		1,183 (15.08)	272 (18.18)	
**Retirement status**, ***N*** **(%)**				< 0.001^‡^			< 0.001^‡^
Non-retirement	3,829 (41.00)	2,544 (52.04)	1,285 (28.88)		3,418 (43.58)	411 (27.47)	
Retirement	5,510 (59.00)	2,345 (47.96)	3,165 (71.12)		4,425 (56.42)	1,085 (72.53)	
**Diet quality, N (%)**				0.001^‡^			0.466^‡^
Unhealthy	2,912 (31.18)	1,452 (29.70)	1,460 (32.81)		2,458 (31.34)	454 (30.35)	
Healthy	6,427 (68.82)	3,437 (70.30)	2,990 (67.19)		5,385 (68.66)	1,042 (69.65)	
**Smoking**, ***N*** **(%)**				0.044^‡^			0.788^‡^
Smoker	1,933 (20.70)	972 (19.88)	961 (21.60)		1,619 (20.64)	314 (20.99)	
Non-Smoker	7,406 (79.30)	3,917 (80.12)	3,489 (78.40)		6,224 (79.36)	1,182 (79.01)	
**Alcohol drinking**, ***N*** **(%)**				0.016^‡^			0.229^‡^
Frequent	566 (6.06)	268 (5.48)	298 (6.70)		486 (6.20)	80 (5.35)	
Never or Occasional	8,773 (93.94)	4,621 (94.52)	4,152 (93.30)		7,357 (93.80)	1,416 (94.65)	
**BMI**, ***N*** **(%)**				< 0.001^‡^			< 0.001^‡^
Unhealthy	5,094 (54.55)	2,312 (47.29)	2,782 (62.52)		4,165 (53.10)	929 (62.10)	
Healthy	4,245 (45.45)	2,577 (52.71)	1,668 (37.48)		3,678 (46.90)	567 (37.90)	
**LTPA**, ***N*** **(%)**				0.622^‡^			0.870^‡^
Unhealthy	1,333 (14.27)	689 (14.09)	644 (14.47)		1,122 (14.31)	211 (14.10)	
Healthy	8,006 (85.73)	4,200 (85.91)	3,806 (85.53)		6,721 (85.69)	1,285 (85.90)	
**Number of dyslipidemia indicators**, ***N*** **(%)**				< 0.001^‡^			< 0.001^‡^
One indicator abnormal	1,946 (20.84)	1,023 (20.92)	923 (20.74)		1,665 (21.23)	281 (18.78)	
Two indicators abnormal	3,816 (40.86)	2,182 (44.63)	1,634 (36.72)		3,343 (42.62)	473 (31.62)	
Three indicators abnormal	2,758 (29.53)	1,348 (27.57)	1,410 (31.69)		2,232 (28.46)	526 (35.16)	
Four indicators abnormal	236 (2.53)	116 (2.37)	120 (2.70)		183 (2.33)	53 (3.54)	
Five indicators abnormal	583 (6.24)	220 (4.50)	363 (8.16)		420 (5.36)	163 (10.90)	

BMI, body mass index; LTPA, leisure-time physical activity; MET-h, metabolic equivalent values-hours; T2DM, type 2 diabetes mellitus. *P value from *t*-test; ^†^*P* value from Wilcoxon rank sum test; ^‡^*P* value Chi-square test.

As shown in Supplementary Table S1, compared with subjects who with neither hypertension nor T2DM, subjects who suffered from hypertension or T2DM or their comorbidity were more likely to be male and non-married, have a lower level of education, be retirees, have an unhealthy diet, and have an unhealthy BMI (all *P* < 0.05). Five individual lifestyle factors were all strongly correlated with HLS (all *P* < 0.001) (Supplementary Table S2).

**Figure 1:**
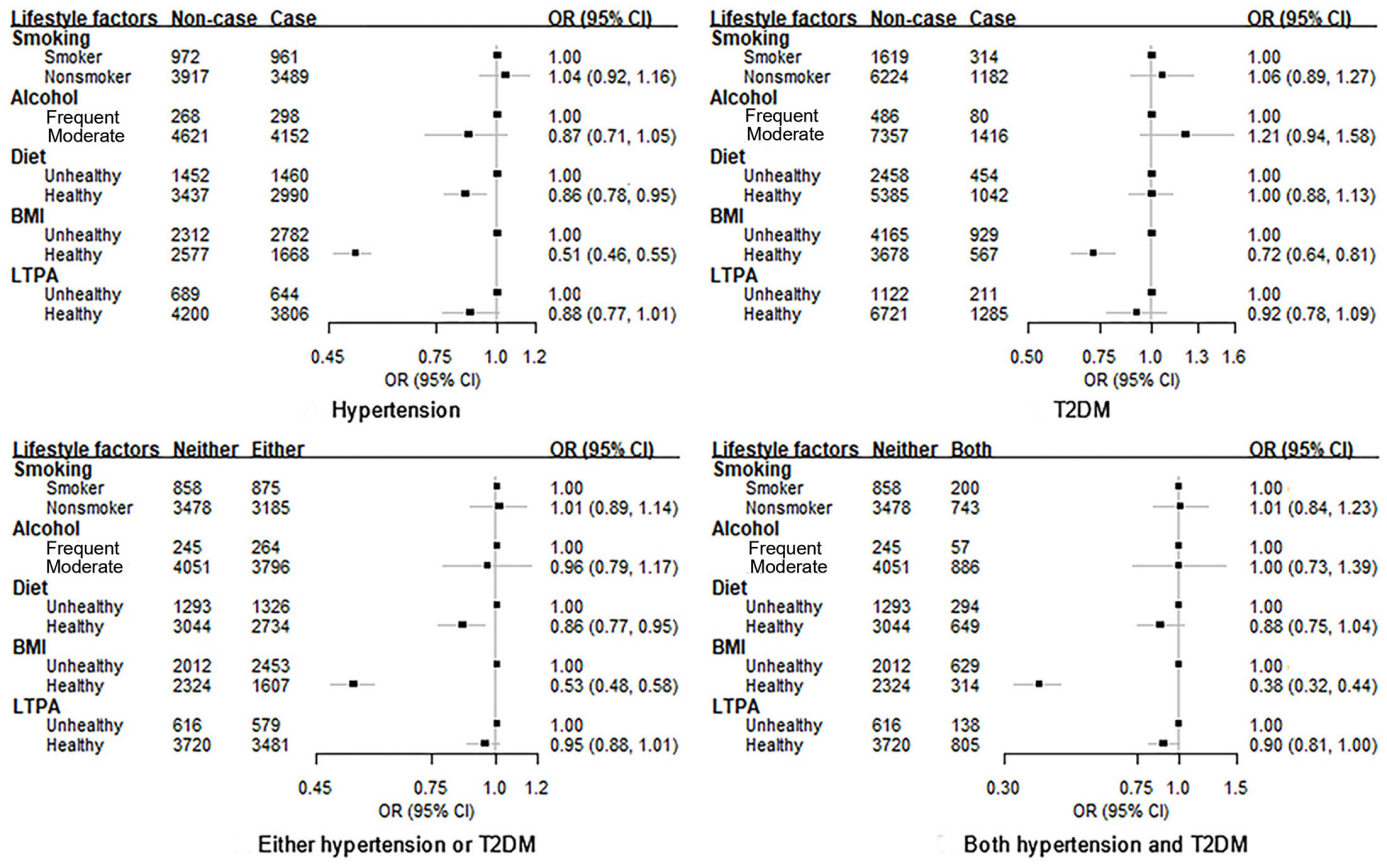
Association between each lifestyle factor and the risk of hypertension, T2DM and their comorbidity among subjects with dyslipidemia. BMI, body mass index; LTPA, leisure-time physical activity; OR, odds radio; CI, confidence interval; T2DM, type 2 diabetes mellitus. Adjust for age, sex, education, marital status, retirement status, diabetes (only for the association with hypertension) or hypertension (only for the association with diabetes), and all other lifestyle factors.

For individual lifestyle factors, as shown in Figure 1 and Supplementary Tables S3–S5, healthy BMI was observed to be associated with a lower risk of hypertension (OR 0.51, 95% CI 0.46**–**0.55) and T2DM (OR 0.72, 95% CI 0.64**–**0.81) respectively after adjusting for confounders, while the healthy diet was only associated with a reduced risk of hypertension (OR 0.86, 95% CI 0.78**–**0.95). When considering hypertension and T2DM simultaneously, similarly, only healthy BMI was associated with a decreased risk of the comorbidity of both hypertension and T2DM (OR: 0.38, 95% CI 0.32–0.44) after considering for confounders.

An increment of HLS was significantly associated with a lower risk of hypertension and T2DM (both *P*__−_trend_ < 0.05) after adjusting for confounders (Table 3). Compared with the subjects with 0**–**2 HLS, the OR for hypertension for subjects with 3, 4, and 5 HLS was 0.86 (95% CI: 0.73–1.01), 0.69 (95% CI: 0.59–0.81), and 0.48 (95% CI: 0.40–0.57), respectively, after adjusting for all confounders. Compared with the subjects with 0**–**2 HLS, the OR for T2DM for subjects with 3, 4, and 5 HLS was 0.85 (95% CI: 0.70–1.05), 0.88 (95% CI: 0.73–1.08), and 0.67 (95% CI: 0.54–0.84), respectively, after adjusting for all confounders. Every 1-point increment of HLS was associated with a 23% (OR: 0.77, 95% CI: 0.73–0.82) decreased risk of hypertension and 11% (OR: 0.89, 95% CI: 0.84–0.96) reduced risk of T2DM.

**Table 3:** Association between healthy lifestyle score and the risk of hypertension and T2DM.

**Healthy lifestyle score**	N*		**OR (95% CI)**		
	**Non-case group**	**Case group**	**Crude OR**	**Adjusted OR^†^**	**Adjusted OR ^‡^**
**Hypertension**					
0–2	496	602	1.00	1.00	1.00
3	1,070	1,175	0.90 (0.78, 1.05)	0.85 (0.72, 0.99)	0.86 (0.73, 1.01)
4	1,941	1,814	0.77 (0.67, 0.88)	0.69 (0.58, 0.81)	0.69 (0.59, 0.81)
5	1,382	859	0.51 (0.44, 0.59)	0.46 (0.39, 0.55)	0.48 (0.40, 0.57)
*P* for trend			< 0.001	< 0.001	< 0.001
Every 1-point increment			0.80 (0.76, 0.83)	0.77 (0.73, 0.81)	0.77 (0.73, 0.82)
**T2DM**					
0–2	906	192	1.00	1.00	1.00
3	1,879	366	0.92 (0.76, 1.11)	0.84 (0.69, 1.03)	0.85 (0.70, 1.05)
4	3,114	641	0.97 (0.81, 1.16)	0.85 (0.70, 1.04)	0.88 (0.73, 1.08)
5	1,944	297	0.72 (0.59, 0.88)	0.62 (0.50, 0.78)	0.67 (0.54, 0.84)
*P* for trend			0.003	< 0.001	0.001
Every 1-point increment			0.92 (0.86, 0.97)	0.87 (0.81, 0.93)	0.89 (0.84, 0.96)

*N represents sample size for non- case group or for case group. ^†^Adjustment for age, sex, education, marital status, and retirement status. ^‡^Additional adjustment for T2DM (for the association with hypertension) or hypertension (for the association with T2DM).

As seen in Table 4, individuals with a higher HLS had a lower risk of suffering from either or both hypertension and T2DM when compared with those being non-hypertension and non-T2DM (both *P*__−_trend_ < 0.05). Compared with the subjects with 0**–**2 HLS, the ORs of suffering from either but not both hypertension and T2DM for subjects with 3, 4, and 5 HLS were 0.82 (95% CI 0.69**–**1.02), 0.71 (95% CI 0.60**–**0.84), and 0.48 (95% CI 0.40**–**0.57) after adjusting for confounders; the ORs of suffering from both hypertension and T2DM for subjects with 3, 4, and 5 HLS were 0.77 (95% CI 0.59**–**0.99), 0.62 (95% CI 0.48**–**0.81), and 0.35 (95% CI 0.26**–**0.47) after adjusting for confounders.

**Table 4:** Association between healthy lifestyle score and the risk of comorbidity of hypertension and T2DM using multi-nominal logistic regression.

**Healthy lifestyle score**	N*			**Crude OR (95% CI)**		**Adjusted OR (95% CI)** ^**†**^	
	**Neither**	**Either**	**Both**	**Either vs. Neither**	**Both vs. Neither**	**Either vs. Neither**	**Both vs. Neither**
0–2	433	536	129	1.00	1.00	1.00	1.00
3	957	1,035	253	0.87 (0.75, 1.02)	0.89 (0.70, 1.13)	0.82 (0.69, 0.97)	0.77 (0.59, 0.99)
4	1,695	1,665	395	0.79 (0.69, 0.92)	0.78 (0.62, 0.98)	0.71 (0.60, 0.84)	0.62 (0.48, 0.81)
5	1,251	824	166	0.53 (0.46, 0.62)	0.45 (0.35, 0.57)	0.48 (0.40, 0.57)	0.35 (0.26, 0.47)
*P* for trend				< 0.001	< 0.001	< 0.001	< 0.001
Every 1-point increment				0.81 (0.78, 0.85)	0.77 (0.71, 0.83)	0.78 (0.74, 0.83)	0.71 (0.65, 0.77)

*N: sample size of total subjects in each healthy lifestyle score group. Neither: subjects with neither hypertension nor T2DM, both: subjects with both hypertension and T2DM; either: subjects with only one disease of the outcome. ^†^Adjustment for age, sex, education, marital status, and retirement status.

When stratified by sex and retirement, a higher HLS was associated with a lower risk of hypertension in each subgroup; however, a higher HLS was significantly associated with a lower risk of T2DM only in females and retirees (Supplementary Tables S6, S7). The multiplicative interactions of HLS with sex (*P*_−interaction_ = 0.005) and retirement (*P*_−interaction_ < 0.001) on hypertension, and with sex (*P*_−interaction_ = 0.033) on T2DM, were observed. Likewise, a higher HLS prevented subjects with dyslipidemia suffering from one or both hypertension and T2DM in both males and females, retirees, and non-retirees (Supplementary Tables S8, S9); stronger associations existed in females and non-retirees (all *P*_−interaction_ < 0.001).

In sensitivity analysis, as seen in Table 5, an increment of HLS was associated with a lower risk of hypertension and T2DM after adjusting for confounders in different dyslipidemia subtypes, however, the association between HLS and T2DM risk was not significant in serum HDL-C < 1.0 mmol/L group and in serum triglyceride ≥ 1.7 mmol/L group. As shown in Table 6, individuals with a higher HLS had a lower risk of suffering from either or both hypertension and T2DM when compared with those being non-hypertension and non- T2DM in different dyslipidemia subtypes (all *P*__−_trend_ < 0.05). Besides, using a weighted lifestyle score did not alter the results remarkably (Supplementary Table S10); repeated analyses also yielded similar results by using the median in place of the WHO-recommended cut-off value of LTPA in generating HLS, by using 25 kg/m^2^ as the cut-off value for healthy and unhealthy BMI in generating HLS, and by replacing BMI with waist circumference as a component of HLS, by excluding subjects with BMI of less than 18.5 kg/m^2^, and by excluding subjects aged 75 years or above, and with additional adjustment for the number of dyslipidemia indicators (Supplementary Tables S11–S17).

**Table 5:** Association between healthy lifestyle score and the risk of hypertension and T2DM according to different dyslipidemia definitions.

**Healthy lifestyle score**	**Hypertension**				**T2DM**			
	N*		**OR (95% CI)**		N*		**OR (95% CI)**	
	**Non-case group**	**Case group**	**Crude OR**	**Adjusted OR^†^**	**Non-case group**	**Case group**	**Crude OR**	**Adjusted OR^†^**
**Self-reported physician-diagnosed dyslipidemia**								
0–2	74	154	1.00	1.00	165	63	1.00	1.00
3	174	373	1.03 (0.74, 1.43)	0.94 (0.65, 1.35)	406	141	0.91 (0.64, 1.29)	0.83 (0.58, 1.20)
4	397	603	0.73 (0.54, 0.99)	0.67 (0.47, 0.96)	752	248	0.86 (0.63, 1.20)	0.80 (0.56, 1.15)
5	315	273	0.42 (0.30, 0.57)	0.39 (0.27, 0.58)	468	120	0.67 (0.47, 0.96)	0.65 (0.44, 0.98)
*P* for trend			< 0.001	< 0.001			< 0.001	0.036
Every 1-point increment			0.70 (0.64, 0.77)	0.70 (0.62, 0.78)			0.70 (0.64, 0.77)	0.88 (0.78, 0.99)
**Serum cholesterol** **≥5.2 mmol/L**								
0–2	349	414	1.00	1.00	633	130	1.00	1.00
3	796	829	0.88 (0.74, 1.04)	0.86 (0.70, 1.04)	1,375	250	0.89 (0.70, 1.12)	0.86 (0.67, 1.10)
4	1,496	1,337	0.75 (0.64, 0.88)	0.69 (0.57, 0.84)	1,372	461	0.95 (0.77, 1.18)	0.90 (0.71, 1.15)
5	1,080	643	0.50 (0.42, 0.60)	0.49 (0.40, 0.61)	1,513	210	0.68 (0.53, 0.86)	0.67 (0.51, 0.87)
*P* for trend			< 0.001	< 0.001			< 0.001	0.006
Every 1-point increment			0.79 (0.75, 0.83)	0.78 (0.73, 0.83)			0.79 (0.75, 0.83)	0.89 (0.82, 0.97)
**Serum LDL-C** **≥3.4 mmol/L**								
0–2	355	423	1.00	1.00	650	128	1.00	1.00
3	811	843	0.87 (0.74, 1.03)	0.85 (0.70, 1.03)	1,405	249	0.90 (0.71, 1.14)	0.85 (0.67, 1.09)
4	1,470	1,314	0.75 (0.64, 0.88)	0.67 (0.55, 0.81)	2,347	437	0.95 (0.76, 1.18)	0.86 (0.68, 1.10)
5	1,041	605	0.49 (0.41, 0.58)	0.46 (0.37, 0.57)	1,439	207	0.73 (0.58, 0.93)	0.69 (0.52, 0.90)
*P* for trend			< 0.001	< 0.001			< 0.001	0.011
Every 1-point increment			0.79 (0.75, 0.83)	0.77 (0.72, 0.82)			0.79 (0.75, 0.83)	0.90 (0.83, 0.98)
**Serum HDL-C** **<** **1.0 mmol/L**								
0–2	87	125	1.00	1.00	166	46	1.00	1.00
3	141	143	0.71 (0.49, 1.01)	0.60 (0.40, 0.89)	221	63	1.03 (0.67, 1.59)	0.93 (0.59, 1.47)
4	191	187	0.68 (0.48, 0.96)	0.55 (0.36, 0.83)	288	90	1.13 (0.76, 1.70)	0.94 (0.59, 1.51)
5	88	61	0.48 (0.31, 0.74)	0.35 (0.20, 0.59)	120	29	0.87 (0.51, 1.46)	0.71 (0.39, 1.29)
*P* for trend			0.001	< 0.001			0.001	0.352
Every 1-point increment			0.81 (0.72, 0.92)	0.73 (0.62, 0.87)			0.81 (0.72, 0.92)	0.92 (0.76, 1.10)
**Serum Triglyceride** **≥1.7 mmol/L**								
0–2	264	345	1.00	1.00	485	124	1.00	1.00
3	479	549	0.88 (0.72, 1.07)	0.80 (0.64, 1.01)	816	212	1.02 (0.79, 1.31)	0.97 (0.75, 1.27)
4	741	848	0.88 (0.73, 1.06)	0.78 (0.62, 0.98)	1,251	338	1.06 (0.84, 1.33)	1.00 (0.77, 1.30)
5	453	326	0.55 (0.44, 0.68)	0.49 (0.37, 0.64)	636	143	0.88 (0.67, 1.15)	0.84 (0.62, 1.14)
*P* for trend			< 0.001	< 0.001			< 0.001	0.319
Every 1-point increment			0.84 (0.79, 0.90)	0.81 (0.75, 0.88)			0.84 (0.79, 0.90)	0.95 (0.87, 1.05)

HDL-C, high-density lipoprotein cholesterol; LDL-C, low-density lipoprotein cholesterol; T2DM, type 2 diabetes mellitus. *N represents sample size for non- case group or for case group. ^†^Adjustment for age, sex, education, marital status, retirement status, and T2DM (for the association with hypertension) or hypertension (for the association with T2DM).

**Table 6:** Association between healthy lifestyle score and the risk of comorbidity of hypertension and T2DM according to different dyslipidemia definitions.

**Healthy lifestyle score**	N*			**Crude OR (95% CI)**		**Adjusted OR (95% CI)** ^**†**^	
	**Neither**	**Either**	**Both**	**Either vs. Neither**	**Both vs. Neither**	**Either vs. Neither**	**Both vs. Neither**
**Self-reported physician-diagnosed dyslipidemia**							
0–2	59	121	48	1.00	1.00	1.00	1.00
3	144	292	111	0.99 (0.68, 1.43)	0.95 (0.60, 1.49)	0.86 (0.58, 1.28)	0.80 (0.49, 1.32)
4	312	525	163	0.82 (0.58, 1.15)	0.64 (0.42, 0.98)	0.70 (0.48, 1.04)	0.55 (0.33, 0.90)
5	269	245	74	0.44 (0.31, 0.63)	0.34 (0.21, 0.54)	0.37 (0.25, 0.57)	0.29 (0.17, 0.50)
*P* for trend				< 0.001	< 0.001	< 0.001	< 0.001
Every 1-point increment				0.73 (0.66, 0.81)	0.65 (0.57, 0.75)	0.69 (0.62, 0.78)	0.63 (0.54, 0.74)
**Serum cholesterol** **≥5.2 mmol/L**							
0–2	307	368	88	1.00	1.00	1.00	1.00
3	719	733	173	0.85 (0.71, 1.02)	0.84 (0.63, 1.12)	0.82 (0.67, 1.00)	0.78 (0.57, 1.06)
4	1,304	1,260	269	0.81 (0.68, 0.96)	0.72 (0.55, 0.94)	0.74 (0.61, 0.90)	0.61 (0.45, 0.84)
5	988	617	118	0.52 (0.43, 0.62)	0.42 (0.31, 0.56)	0.49 (0.39, 0.61)	0.36 (0.25, 0.52)
*P* for trend				< 0.001	< 0.001	< 0.001	< 0.001
Every 1-point increment				0.81 (0.77, 0.86)	0.75 (0.69, 0.82)	0.79 (0.74, 0.84)	0.71 (0.64, 0.79)
**Serum LDL-C** **≥3.4 mmol/L**							
0–2	314	377	87	1.00	1.00	1.00	1.00
3	735	746	173	0.85 (0.71, 1.01)	0.85 (0.64, 1.13)	0.80 (0.66, 0.98)	0.77 (0.57, 1.06)
4	1,293	1,231	260	0.79 (0.67, 0.94)	0.73 (0.55, 0.95)	0.69 (0.57, 0.84)	0.59 (0.43, 0.80)
5	948	584	114	0.51 (0.43, 0.62)	0.43 (0.32, 0.59)	0.46 (0.37, 0.57)	0.36 (0.25, 0.51)
*P* for trend				< 0.001	< 0.001	< 0.001	< 0.001
Every 1-point increment				0.81 (0.77, 0.85)	0.76 (0.70, 0.83)	0.77 (0.72, 0.83)	0.70 (0.63, 0.78)
**Serum HDL-C** **<** **1.0 mmol/L**							
0–2	76	101	35	1.00	1.00	1.00	1.00
3	116	130	38	0.84 (0.57, 1.24)	0.71 (0.41, 1.23)	0.77 (0.51, 1.17)	0.51 (0.28, 0.94)
4	156	167	55	0.81 (0.56, 1.17)	0.77 (0.46, 1.27)	0.72 (0.46, 1.11)	0.48 (0.26, 0.90)
5	74	60	15	0.61 (0.39, 0.96)	0.44 (0.22, 0.87)	0.50 (0.29, 0.86)	0.23 (0.10, 0.51)
*P* for trend				0.042	0.044	0.019	0.001
Every 1-point increment				0.87 (0.76, 0.99)	0.82 (0.67, 0.99)	0.82 (0.69, 0.97)	0.66 (0.52, 084)
Serum Triglyceride ≥ 1.7 mmol/L							
0–2	225	299	85	1.00	1.00	1.00	1.00
3	408	479	141	0.88 (0.71, 1.10)	0.91 (0.67, 1.25)	0.82 (0.65, 1.04)	0.80 (0.56, 1.13)
4	615	762	212	0.93 (0.76, 1.14)	0.91 (0.68, 1.22)	0.85 (0.67, 1.09)	0.77 (0.54, 1.09)
5	382	325	72	0.64 (0.51, 0.80)	0.50 (0.35, 0.71)	0.58 (0.44, 0.77)	0.40 (0.26, 0.61)
*P* for trend				< 0.001	< 0.001	< 0.001	< 0.001
Every 1-point increment				0.89 (0.83, 0.95)	0.83 (0.75, 0.92)	0.86 (0.79, 0.93)	0.77 (0.68, 0.87)

HDL-C, high-density lipoprotein cholesterol; LDL-C, low-density lipoprotein cholesterol; T2DM, type 2 diabetes mellitus. *N: sample size of total subjects in each healthy lifestyle score group. Neither: subjects with neither hypertension nor T2DM, both: subjects with both hypertension and T2DM; either: subjects with only one disease of the outcome. ^†^Adjustment for age, sex, education, marital status, and retirement status.

## Discussion

To the best of our knowledge, this is the first large-scale population-based study conducted in China to investigate both individual and combined effects of healthy lifestyle factors on the risk of hypertension and T2DM among subjects with dyslipidemia. Our study found that HLS established by five main modifiable lifestyle factors, including smoking, alcohol drinking, diet, BMI, and LTPA, was adversely associated with the risk of hypertension, T2DM, and their commodity among subjects with dyslipidemia. Consistent results were also yielded in a series of sensitivity analyses, highlighting the importance of adopting a healthy lifestyle to prevent hypertension and diabetes among the populations with dyslipidemia.

Our results are broadly consistent with previous studies conducted among the general population (11, 12, 14, 29–33). Although lifestyle factors included in these studies were slightly different, findings of all studies consistently revealed beneficial effects on hypertension and diabetes by adopting healthy lifestyles. In two prospective cohort studies of women (29, 30) and a prospective cohort study of men (11), adherence to more healthy lifestyle factors was associated with a decreased risk of self-reported hypertension. A prospective cohort study in Australia also reported that having a higher number of lifestyle risk factors (i.e., high BMI, high alcohol intake, low physical activity levels, current smoking, low vegetable and fruit intake, and high risk of psychological distress) was associated with a higher risk of self-reported hypertension among middle-aged and older adults (12). A recent report from the Hortega Study found that adherence to 3**–**5 healthy lifestyle factors, relative to those with 0**–**1 healthy lifestyle factors, showed an 80% decreased risk of T2DM (32). In a cohort study in Spain, healthy lifestyle behaviors, including traditional modified lifestyle factors and other lifestyle indicators not typically included in risk scores, were associated with a 46% relative decreased hazard of T2DM (33). In addition, our results show that adherence to a healthier lifestyle is associated with a reduced risk of one or both comorbidities of hypertension and type 2 diabetes in subjects with dyslipidemia, and the effect is more pronounced in subjects with comorbidities. Our results for the first time indicated that even in the subjects with dyslipidemia, adherence to healthier lifestyle behaviors still exerts beneficial effects on preventing hypertension and T2DM and needs to be encouraged.

Our study showed that among the subjects with dyslipidemia, a healthy BMI was still a significant protective factor for hypertension, T2DM, and their comorbidity. A retrospective cross-sectional study involving 90,047 adults aged 18**–**85 years indicated that a higher BMI was associated with an increased prevalence of hypertension, diabetes, and dyslipidemia in both Japan and the USA (34). Another study conducted in China also found that overweight and obese individuals had a significantly higher risk than normal-weight people to develop hypertension, and dyslipidemia significantly shared interactions with overweight and obesity that increased the risk of hypertension (35). A meta-analysis including 18 prospective cohort studies showed that obesity and overweight were associated with about 7-fold risk and 3-fold risk of diabetes (36). Compared with subjects with healthy BMI, overweight and obese individuals are more likely to suffer metabolism disorders and insulin resistance, which may lead to the occurrence of hypertension and diabetes (35). Hence, sustained weight loss may be the primary driver of decreased risk of hypertension, diabetes, and other cardiometabolic diseases in the long term (37, 38). We also found that a healthy diet was associated with a lower risk of hypertension and of suffering from either hypertension or T2DM in the subjects with dyslipidemia. A previous study from NHS showed that adopting a low-risk diet was associated with a significantly lower incidence of hypertension among general women (29). However, we did not find an apparent association between other lifestyle factors with the risk of hypertension, T2DM, or their comorbidity. Similar results were also reported in the previous studies (39, 40). Even so, this current study showed that HLS was associated with a decreased risk of hypertension, T2DM, and their comorbidity, which may be due to that the beneficial effect of different components of HLS is synergistic and cumulative, and the synergistic association of these factors became greater than individual effect. Our findings highlighted the importance of a combined health effect of different lifestyle behaviors for the prevention of hypertension and T2DM among subjects with dyslipidemia, instead of just concerning a single lifestyle choice.

One interesting result of the present study was that the negative associations between HLS and the risk of hypertension, T2DM, and suffering from one or both hypertension and T2DM were stronger in females than in males, which was consistent with previous reports (12, 41). The reason for this difference may be due to physiologic changes related to aging, changes in sex hormones, increased arterial stiffness, and lower responsiveness of the sympathetic nervous system (30, 42, 43). We also found a stronger association of HLS with the risk of hypertension and suffering from one or both hypertension and T2DM in non-retirees than in retirees. The physical damage from years of work was unavoidable, which may negatively affect retirees' physical function in their old age (44).

There are several strengths in our study. First, this is the first large-scale population-based study among Chinese adults to explore the association of combined effects of healthy lifestyle factors with the risk of hypertension, T2DM, and their comorbidity in the subjects with dyslipidemia. Our findings will provide useful clues for further prospective studies. Second, subjects in the present study were recruited by using the multistage sampling method, which can potentiate the representativeness of subjects and minimize selection bias to some degree. Moreover, the questionnaire survey was conducted face to face by trained medical workers, which can to some degree reduce the information bias. Third, a series of sensitivity analyses were carried out and consistent results were displayed, which indicated a relatively good internal consistency for the HLS assessed.

Some limitations also exist in this study. First, this study was a cross-sectional study, which limited the ability to determine the direction of the association and infer a causal association. Second, the diagnosis of T2DM was determined by fasting blood glucose and self-report, but a lack of oral glucose tolerance test and determination of glycosylated hemoglobin may cause a few subjects with diabetes to not be identified; however, this would not lead to a fundamental change in the results of the study. Third, dietary information over the past 12 months was collected from each subject using the FFQ, which might result in recall bias inevitably. However, face-to-face questionnaire surveys and physical examinations were conducted at baseline by trained medical workers, which can to a large degree reduce the bias. Finally, although our study adjusted for several possible confounders, there may be residual confounding due to unmeasured factors, such as genetic factors. However, GZHS is an ongoing cohort study, possible confounding factors will be added in future research to verify our study.

## Conclusions

In summary, the results suggest that the more kinds of healthy lifestyle, the lower the risk of hypertension, T2DM, and their comorbidity among subjects with dyslipidemia. Preventive strategies integrating multiple-dimensional lifestyle factors may provide a more feasible approach for the prevention and management of main chronic diseases.

## Data availability statement

The raw data supporting the conclusions of this article will be made available by the authors, without undue reservation.

## Ethics statement

The studies involving human participants were reviewed and approved by the Ethical Review Committee for Biomedical Research, School of Public Health, Sun Yat-sen University. The patients/participants provided their written informed consent to participate in this study.

## Author contributions

XL and HD conceived the study. XL, WZ, and HD supervised the study. MZ, JH, XD, MX, YZ, YL, and LL collected the data. PH analyzed the data. PH, MZ, and XD drafted the manuscript. WZ, HZ, HD, and XL reviewed and edited the manuscript. All authors provided comments and approved the final version.

## Funding

This work was supported by the Science and Technology Program of Guangzhou City (No. 202102080404), the Guangdong Basic and Applied Basic Research Foundation (No. 2022A1515010686), the Guangdong Provincial Key R&D Program (No. 2019B020230004), and the National Key R&D Program of China (No. 2018YFC1312502).

## Conflict of interest

The authors declare that the research was conducted in the absence of any commercial or financial relationships that could be construed as a potential conflict of interest.

## Publisher's note

All claims expressed in this article are solely those of the authors and do not necessarily represent those of their affiliated organizations, or those of the publisher, the editors and the reviewers. Any product that may be evaluated in this article, or claim that may be made by its manufacturer, is not guaranteed or endorsed by the publisher.

## Abbreviations

BMI: body mass index
CI: confidence interval
FFQ: food frequency questionnaire
HLS: healthy lifestyle score
LTPA: leisure-time physical activity
OR: odds ratio
T2DM: type 2 diabetes mellitus.
