# Supplementary material for: Association of healthy lifestyles on the risk of hypertension, type 2 diabetes mellitus, and their comorbidity among subjects with dyslipidemia
Source: Front Nutr. 2022 Sep 26;9:1006379. doi: 10.3389/fnut.2022.1006379 (PMC9550234; doi:10.3389/fnut.2022.1006379)
Supplement: Supplementary file 1 [file Table_1.docx]

**Supplementary Material**

Figure S1 Flow chart of selection of subjects with dyslipidemia

Table S1 Baseline characteristic of the subjects by comorbidity status

Table S2 The correlation coefficients between each lifestyle factor and healthy lifestyle score

Table S3 Association between each lifestyle score and hypertension risk

Table S4 Association between each lifestyle factor and T2DM risk

Table S5 Association between each lifestyle factor and the risk of risk of comorbidity of hypertension and T2DM

Table S6 Stratified analysis on the association between healthy lifestyle score and the risk of hypertension and T2DM by sex

Table S7 Stratified analysis on the association between healthy lifestyle score and the risk of hypertension and T2DM by retirement status

Table S8 Stratified analysis on the association between healthy lifestyle score and the risk of comorbidity of hypertension and T2DM by sex

Table S9 Stratified analysis on the association between healthy lifestyle score and the risk of comorbidity of hypertension and T2DM by retirement status.

Table S10 Sensitivity analyses on the association between healthy lifestyle score and the risk of hypertension and T2DM using weighted lifestyle score

Table S11 Sensitivity analyses on the association of healthy lifestyle score and the risk of comorbidity status using multinominal logistic regression

Table S12 Sensitivity analyses on the association between healthy lifestyle score and the risk of hypertension and T2DM by using median of LTPA in generating HLS

Table S13 Sensitivity analyses on the association between healthy lifestyle score and the risk of hypertension and T2DM by using BMI=25 kg/m2 as cut-off value in generating HLS

Table S14 Sensitivity analyses on the association between healthy lifestyle score and the risk of hypertension and T2DM after replacing the BMI with waist circumference

Table S15 Sensitivity analyses on the association between healthy lifestyle score and the risk of hypertension and T2DM after excluding participants with BMI less than 18.5 kg/m^2^

Table S16 Sensitivity analyses on the association between healthy lifestyle score and the risk of hypertension and T2DM after excluding participants aged 75 years and above

Table S17 Association between healthy lifestyle score and the risk of hypertension and T2DM with additional adjustment for the number of dyslipidemia indicators

Figure S1 Flow chart of selection of subjects with dyslipidemia

Original dataset of the Guangzhou Heart Study

(12,013 subjects)

9,339 subjects with dyslipidemia were included in the study

- Excluding subjects with missing information on the blood pressure or diabetes-related information (n=5)
- Excluding subjects without dyslipidemia (n=2,669)

**Table S1 Baseline characteristic of the subjects by comorbidity status**

| **Characteristic** | **Total**  **(N=9339)** | **Neither hypertension nor diabetes (N=4336)** | **Either Hypertension or T2DM (N=4060)** | **Both hypertension and T2DM (N=943)** | ***P value*** |
| --- | --- | --- | --- | --- | --- |
| Age, years, mean (S.D.) | 59.49 (11.44) | 55.04 (10.49) | 62.61 (10.87) | 66.53 (9.97) | <0.001^*^ |
| BMI, kg/m^2^, mean (S.D.) | 24.21 (3.54) | 23.32 (3.24) | 24.85 (3.58) | 25.50 (3.68) | <0.001^*^ |
| LTPA, MET-h, median (Interquartile) | 34.65 (41.10) | 34.65 (42.46) | 35.70 (40.40) | 34.65 (37.24) | 0.606^†^ |
| Diet quality score, mean (S.D.) | 7.403 (1.80) | 7.444 (1.782) | 7.349 (1.827) | 7.442 (1.79) | 0.210^*^ |
| Sex, N (%) |  |  |  |  | <0.001^‡^ |
| Male | 3228 (34.56) | 1363 (31.43) | 1505 (37.07) | 360 (38.18) |  |
| Female | 6111 (65.44) | 2973 (68.57) | 2555 (62.93) | 583 (61.82) |  |
| Education, N (%) |  |  |  |  | <0.001^‡^ |
| < high school | 5809 (62.20) | 2483 (57.26) | 2680 (66.01) | 646 (68.50) |  |
| ≥ high school | 3530 (37.80) | 1853 (42.74) | 1380 (33.99) | 297 (31.50) |  |
| Material status, N (%) |  |  |  |  | <0.001^‡^ |
| Married | 7884 (84.42) | 3794 (87.50) | 3343 (82.34) | 747 (79.22) |  |
| Others | 1455 (15.58) | 542 (12.50) | 717 (17.66) | 196 (20.78) |  |
| Retirement status, N (%) |  |  |  |  | <0.001^‡^ |
| Non-retirement | 3829 (41.00) | 2323 (53.57) | 1316 (32.41) | 190 (20.15) |  |
| Retirement | 5510 (59.00) | 2013 (46.43) | 2744 (67.59) | 753 (79.85) |  |
| Diet quality, N (%) |  |  |  |  | 0.018^‡^ |
| Unhealthy | 2912 (31.18) | 1292 (29.80) | 1326 (32.66) | 294 (31.18) |  |
| Healthy | 6427 (68.82) | 3044 (70.20) | 2734 (67.34) | 649 (68.82) |  |
| Smoking, N (%) |  |  |  |  | 0.126^‡^ |
| Smoker | 1933 (20.70) | 858 (19.79) | 875 (21.55) | 200 (21.21) |  |
| Nonsmoker | 7406 (79.30) | 3478 (80.21) | 3185 (78.45) | 743 (78.79) |  |
| Alcohol drinking, N (%) |  |  |  |  | 0.263^‡^ |
| Drinker | 566 (6.06) | 245 (5.65) | 264 (6.50) | 57 (6.04) |  |
| Nondrinker | 8773 (93.94) | 4091 (94.35) | 3796 (93.50) | 886 (93.96) |  |
| BMI, N (%) |  |  |  |  | <0.001^‡^ |
| Unhealthy | 5094 (54.55) | 2012 (46.40) | 2453 (60.42) | 629 (66.70) |  |
| Healthy | 4245 (45.45) | 2324 (53.60) | 1607 (39.58) | 314 (33.30) |  |
| LTPA, N (%) |  |  |  |  | 0.943^‡^ |
| Unhealthy | 1333 (14.27) | 616 (14.21) | 579 (14.26) | 138 (14.63) |  |
| Healthy | 8006 (85.73) | 3720 (85.79) | 3481 (85.74) | 805 (85.37) |  |
| Dyslipidemia subtypes, N (%) |  |  |  |  | <0.001^‡^ |
| One indicator abnormal | 1946 (20.80) | 927 (21.40) | 834 (20.50) | 185 (19.60) |  |
| Two indicators abnormal | 3816 (40.90) | 1995 (46.00) | 1535 (37.80) | 286 (30.30) |  |
| Three indicators abnormal | 2758 (29.50) | 1148 (26.50) | 1284 (31.60) | 326 (34.60) |  |
| Four indicators abnormal | 236 (2.50) | 96 (2.20) | 107 (2.60) | 33 (3.50) |  |
| Five indicators abnormal | 583 (6.20) | 170 (3.90) | 300 (7.40) | 113 (12.00) |  |

Abbreviations: BMI, body mass index; LTPA, leisure-time physical activity; MET-h, metabolic equivalent values-hours; T2DM, type 2 diabetes mellitus.

* P values of continuous variables were from one-way analysis of variance;

^†^ *P* values of leisure-time physical activity were from Kruskal-Wallis rank sum test;

^‡^*P* values of categorical variables were from chi-square tests.

**Table S2 The correlation coefficients between each lifestyle factor and healthy lifestyle score**

| Lifestyle factor | r_s_^*^ | *P* |
| --- | --- | --- |
| Smoking | 0.523 | <0.001 |
| Alcohol drinking | 0.328 | <0.001 |
| Diet | 0.584 | <0.001 |
| Body mass index | 0.563 | <0.001 |
| Leisure-time physical activity | 0.439 | <0.001 |

^*^ r_s_ represents the Spearman correlation coefficient between individual lifestyle factor and healthy lifestyle score.

**Table S3 Association between each lifestyle factor and hypertension risk**

|  | N^*^ | |  | Effect | | |
| --- | --- | --- | --- | --- | --- | --- |
|  | Non-hypertension group | Hypertension group |  | Unadjusted OR  (95% CI) | Adjusted OR  (95% CI) ^†^ | Adjusted OR  (95% CI) ^‡^ |
| Smoking |  |  |  |  |  |  |
| Smoker | 972 | 961 |  | 1.00 | 1.00 | 1.00 |
| Nonsmoker | 3917 | 3489 |  | 0.90 (0.82, 0.99) | 1.03 (0.92, 1.16) | 1.04 (0.92, 1.16) |
| Alcohol drinker |  |  |  |  |  |  |
| Frequent | 268 | 298 |  | 1.00 | 1.00 | 1.00 |
| Moderate | 4621 | 4152 |  | 0.81 (0.68, 0.96) | 0.88 (0.72, 1.07) | 0.87 (0.71, 1.05) |
| Diet |  |  |  |  |  |  |
| Unhealthy | 1452 | 1460 |  | 1.00 | 1.00 | 1.00 |
| Healthy | 3437 | 2990 |  | 0.87 (0.79, 0.94) | 0.86 (0.78, 0.95) | 0.86 (0.78, 0.95) |
| Body mass index |  |  |  |  |  |  |
| Unhealthy | 2312 | 2782 |  | 1.00 | 1.00 | 1.00 |
| Healthy | 2577 | 1668 |  | 0.54 (0.50, 0.58) | 0.50 (0.45, 0.54) | 0.51 (0.46, 0.55) |
| Leisure-time physical activity |  |  |  |  |  |  |
| Unhealthy | 689 | 644 |  | 1.00 | 1.00 | 1.00 |
| Healthy | 4200 | 3806 |  | 0.97 (0.86, 1.09) | 0.88 (0.77, 1.01) | 0.88 (0.77, 1.01) |

^*^ N represents sample size for non- hypertension group or for hypertension group.

^†^ Adjustment for age, sex, education, marital status, retirement status.

^‡^ Additional adjustment for T2DM.

**Table S4 Association between each lifestyle factor and T2DM risk**

|  | N^*^ | |  | Effect | | |
| --- | --- | --- | --- | --- | --- | --- |
|  | Non- T2DM group | T2DM group |  | Unadjusted OR (95% CI) | Adjusted OR (95% CI) ^†^ | Adjusted OR (95% CI) ^‡^ |
| Smoking |  |  |  |  |  |  |
| Smoker | 1619 | 314 |  | 1.00 | 1.00 | 1.00 |
| Nonsmoker | 6224 | 1182 |  | 0.98 (0.86, 1.12) | 1.10 (0.92, 1.31) | 1.06 (0.89, 1.27) |
| Alcohol drinker |  |  |  |  |  |  |
| Frequent | 486 | 80 |  | 1.00 | 1.00 | 1.00 |
| Moderate | 7357 | 1416 |  | 1.17 (0.92, 1.50) | 1.20 (0.93, 1.55) | 1.21 (0.94, 1.58) |
| Diet |  |  |  |  |  |  |
| Unhealthy | 2458 | 454 |  | 1.00 | 1.00 | 1.00 |
| Healthy | 5385 | 1042 |  | 1.05 (0.93, 1.18) | 0.98 (0.87, 1.11) | 1.00 (0.88, 1.13) |
| Body mass index |  |  |  |  |  |  |
| Unhealthy | 4165 | 929 |  | 1.00 | 1.00 | 1.00 |
| Healthy | 3678 | 567 |  | 0.69 (0.62, 0.77) | 0.67 (0.60, 0.76) | 0.72 (0.64, 0.81) |
| Leisure-time physical activity |  |  |  |  |  |  |
| Unhealthy | 1122 | 211 |  | 1.00 | 1.00 | 1.00 |
| Healthy | 6721 | 1285 |  | 1.02 (0.87, 1.19) | 0.91 (0.77, 1.08) | 0.92 (0.78, 1.09) |

^*^ N represents sample size for non- diabetes group or for T2DM group.

^†^ Adjustment for age, sex, education, marital status, retirement status.

^‡^ Additional adjustment for hypertension.

**Table S5 Association between each lifestyle factor and the risk of** **risk of comorbidity of hypertension and T2DM**

| Healthy lifestyle score | N^*^ | | |  | Crude OR (95% CI) | |  | Adjusted OR (95% CI) ^†^ | |
| --- | --- | --- | --- | --- | --- | --- | --- | --- | --- |
|  | Neither | Either | both |  | Either vs Neither | Both vs Neither |  | Either vs Neither | Both vs Neither |
| Smoking |  |  |  |  |  |  |  |  |  |
| Smoker | 858 | 875 | 200 |  | 1.00 | 1.00 |  | 1.00 | 1.00 |
| Nonsmoker | 3478 | 3185 | 743 |  | 0.90 (0.81, 0.99) | 0.92 (0.77, 1.09) |  | 1.01 (0.89, 1.14) | 1.01 (0.84, 1.23) |
| Alcohol |  |  |  |  |  |  |  |  |  |
| Frequent | 245 | 264 | 57 |  | 1.00 | 1.00 |  | 1.00 | 1.00 |
| Moderate | 4091 | 3796 | 886 |  | 0.86 (0.72, 1.03) | 0.93 (0.69, 1.25) |  | 0.96 (0.79, 1.17) | 1.00 (0.73, 1.39) |
| Diet |  |  |  |  |  |  |  |  |  |
| Unhealthy | 1292 | 1326 | 294 |  | 1.00 | 1.00 |  | 1.00 | 1.00 |
| Healthy | 3044 | 2734 | 649 |  | 0.88 (0.80, 0.96) | 0.94 (0.80, 1.09) |  | 0.86 (0.77, 0.95) | 0.88 (0.75, 1.04) |
| Body mass index |  |  |  |  |  |  |  |  |  |
| Unhealthy | 2012 | 2453 | 629 |  | 1.00 | 1.00 |  | 1.00 | 1.00 |
| Healthy | 2324 | 1607 | 314 |  | 0.57 (0.52, 0.62) | 0.43 (0.37, 0.50) |  | 0.53 (0.48, 0.58) | 0.38 (0.32, 0.44) |
| Leisure-time physical activity |  |  |  |  |  |  |  |  |  |
| Unhealthy | 616 | 579 | 138 |  | 1.00 | 1.00 |  | 1.00 | 1.00 |
| Healthy | 3720 | 3481 | 805 |  | 0.99 (0.88, 1.13) | 0.97 (0.79, 1.18) |  | 0.95 (0.88, 1.01) | 0.90 (0.81, 1.00) |

^*^ N: sample size of total subjects in each healthy lifestyle score group. Neither: subjects with neither hypertension nor T2DM, both: subjects with both hypertension and T2DM; either: subjects with only one disease of outcome.

^†^ Adjustment for age, sex, education, marital status, retirement status and all other lifestyle factors.

**Table S6 Stratified analysis on the association between healthy lifestyle score and the risk of hypertension and T2DM by sex**

| Healthy lifestyle score | Male | | | |  | Female | | | |
| --- | --- | --- | --- | --- | --- | --- | --- | --- | --- |
|  | Non-case group | Case group | Crude OR (95% CI) | Adjusted OR (95% CI) ^*^ |  | Non-case group | Case group | Crude OR (95% CI) | Adjusted OR (95% CI) ^*^ |
| Hypertension (*P*-interaction=0.005) | | | | | | | | | |
| 0-2 | 421 | 514 | 1.00 | 1.00 |  | 75 | 88 | 1.00 | 1.00 |
| 3 | 522 | 533 | 0.84 (0.70, 0.99) | 0.75 (0.62, 0.91) |  | 548 | 642 | 1.00 (0.72, 1.39) | 1.31 (0.90, 1.90) |
| 4 | 461 | 469 | 0.83 (0.69, 0.99) | 0.66 (0.54, 0.81) |  | 1480 | 1345 | 0.77 (0.56, 1.06) | 0.97 (0.68, 1.28) |
| 5 | 151 | 157 | 0.86 (0.66, 1.10) | 0.62 (0.47, 0.82) |  | 1231 | 702 | 0.49 (0.35, 0.67) | 0.63 (0.44, 0.91) |
| *P* for trend |  |  | 0.081 | <0.001 |  |  |  | <0.001 | <0.001 |
| Every 1-point increment |  |  | 0.94 (0.87, 1.01) | 0.84 (0.78, 0.91) |  |  |  | 0.72 (0.67, 0.77) | 0.73 (0.68, 0.79) |
| T2DM (*P*-interaction=0.033) | | | | | | | | | |
| 0-2 | 775 | 160 | 1.00 | 1.00 |  | 131 | 32 | 1.00 | 1.00 |
| 3 | 897 | 158 | 0.85 (0.67, 1.08) | 0.76 (0.59, 0.98) |  | 982 | 208 | 0.87 (0.58, 1.33) | 0.92 (0.61, 1.43) |
| 4 | 756 | 174 | 1.11 (0.88, 1.41) | 0.94 (0.73, 1.21) |  | 2358 | 467 | 0.81 (0.55, 1.23) | 0.85 (0.57, 1.30) |
| 5 | 248 | 60 | 1.17 (0.84, 1.62) | 0.92 (0.65, 1.30) |  | 1696 | 237 | 0.57 (0.38, 0.87) | 0.62 (0.41, 0.95) |
| *P* for trend |  |  | <0.001 | 0.889 |  |  |  | <0.001 | <0.001 |
| Every 1-point increment |  |  | 0.82 (0.75, 0.89) | 0.99 (0.90, 1.10) |  |  |  | 0.82 (0.75, 0.89) | 0.83 (0.76, 0.91) |

^*^ Adjustment for age, education, marital status, retirement status and diabetes (for the association with hypertension) or hypertension (for the association with diabetes).

**Table S7 Stratified analysis on the association between healthy lifestyle score and the risk of hypertension and T2DM by retirement status**

| Healthy lifestyle score | Non-retirement | | | |  | Retirement | | | |
| --- | --- | --- | --- | --- | --- | --- | --- | --- | --- |
|  | Non-case group | Case group | Crude OR (95% CI) | Adjusted OR (95% CI) ^*^ |  | Non-case group | Case group | Crude OR (95% CI) | Adjusted OR (95% CI) ^*^ |
| Hypertension (*P*-interaction<0.001) | | | | | | | | | |
| 0-2 | 334 | 303 | 1.00 | 1.00 |  | 162 | 299 | 1.00 | 1.00 |
| 3 | 655 | 382 | 0.64 (0.53, 0.79) | 0.71 (0.56, 0.88) |  | 415 | 793 | 1.04 (0.83, 1.30) | 1.16 (0.91, 1.47) |
| 4 | 948 | 458 | 0.53 (0.44, 0.64) | 0.62 (0.49, 0.77) |  | 993 | 1356 | 0.74 (0.60, 0.91) | 0.89 (0.70, 1.12) |
| 5 | 607 | 142 | 0.26 (0.20, 0.33) | 0.33 (0.25, 0.44) |  | 775 | 717 | 0.50 (0.40, 0.62) | 0.67 (0.52, 0.85) |
| *P* for trend |  |  | <0.001 | <0.001 |  |  |  | <0.001 | <0.001 |
| Every 1-point increment |  |  | 0.67 (0.63, 0.72) | 0.72 (0.66, 0.79) |  |  |  | 0.75 (0.71, 0.80) | 0.82 (0.76, 0.88) |
| T2DM (*P*-interaction=0.394) | | | | | | | | | |
| 0-2 | 550 | 87 | 1.00 | 1.00 |  | 356 | 105 | 1.00 | 1.00 |
| 3 | 917 | 120 | 0.83 (0.62, 1.11) | 0.91 (0.66, 1.24) |  | 962 | 246 | 0.87 (0.67, 1.13) | 0.84 (0.64, 1.10) |
| 4 | 1265 | 141 | 0.70 (0.53, 0.94) | 0.82 (0.60, 1.14) |  | 1849 | 500 | 0.92 (0.72, 1.17) | 0.92 (0.71, 1.20) |
| 5 | 686 | 63 | 0.58 (0.41, 0.82) | 0.73 (0.49, 1.09) |  | 1258 | 234 | 0.63 (0.49, 0.82) | 0.66 (0.50, 0.88) |
| *P* for trend |  |  | <0.001 | 0.105 |  |  |  | <0.001 | 0.008 |
| Every 1-point increment |  |  | 0.84 (0.75, 0.93) | 0.90 (0.80, 1.02) |  |  |  | 0.87 (0.81, 0.94) | 0.90 (0.83, 0.97) |

^*^ Adjustment for age, sex, education, marital status and T2DM (for the association with hypertension) or hypertension (for the association with T2DM).

**Table S8 Stratified analysis on the association between healthy lifestyle score and the risk of comorbidity of hypertension and T2DM by sex**

|  | Male | | | | | |  | Female | | | | | |
| --- | --- | --- | --- | --- | --- | --- | --- | --- | --- | --- | --- | --- | --- |
|  | N^*^ | | |  | OR (95% CI) ^†^ | |  | N^*^ | | |  | OR (95% CI) ^†^ | |
|  | Neither | Either | Both |  | Either vs Neither | Both vs Neither ^†^ |  | Neither | Either | Both |  | Either vs Neither | Both vs Neither ^†^ |
| 0-2 | 367 | 462 | 106 |  | 1.00 | 1.00 |  | 66 | 74 | 23 |  | 1.00 | 1.00 |
| 3 | 472 | 475 | 108 |  | 0.70 (0.57, 0.85) | 0.62 (0.45, 0.85) |  | 485 | 560 | 145 |  | 1.27 (0.87, 1.87) | 1.19 (0.68, 2.07) |
| 4 | 396 | 425 | 109 |  | 0.68 (0.55, 0.84) | 0.64 (0.46, 0.89) |  | 1299 | 1240 | 286 |  | 0.99 (0.68, 1.43) | 0.81 (0.47, 1.38) |
| 5 | 128 | 143 | 37 |  | 0.65 (0.48, 0.88) | 0.59 (0.37, 0.93) |  | 1123 | 681 | 129 |  | 0.62 (0.43, 0.91) | 0.41 (0.24, 0.72) |
| P for trend |  |  |  |  | <0.001 | 0.009 |  |  |  |  |  | <0.001 | <0.001 |
| Every 1-point increment |  |  |  |  | 0.86 (0.79, 0.93) | 0.84 (0.74, 0.96) |  |  |  |  |  | 0.73 (0.68, 0.79) | 0.63 (0.56, 0.71) |

^*^ N: sample size of total subjects in each healthy lifestyle score group. Neither: subjects with neither hypertension nor T2DM, both: subjects with both hypertension and T2DM; either: subjects with only one disease of outcome.

^†^ OR (95% CI) was calculated by using multinominal logistic regression and with adjustment for age, education, marital status, and retirement status.

^‡^ *P*_-interaction_ (Either vs Neither) = 0.001; *P*_-interaction_ (Both vs Neither) = 0.023.

**Table S9 Stratified analysis on the association between healthy lifestyle score and the risk of comorbidity of hypertension and T2DM by retirement status**

|  | Non-retirement | | | | | |  | Retirement | | | | | |
| --- | --- | --- | --- | --- | --- | --- | --- | --- | --- | --- | --- | --- | --- |
|  | N^*^ | | |  | OR (95% CI) ^†^ | |  | N^*^ | | |  | OR (95% CI) ^†^ | |
|  | Neither | Either | Both |  | Either vs Neither | Both vs Neither ^†^ |  | Neither | Either | Both |  | Either vs Neither | Both vs Neither ^†^ |
| 0-2 | 637 | 282 | 54 |  | 1.00 | 1.00 |  | 461 | 254 | 75 |  | 1.00 | 1.00 |
| 3 | 1037 | 360 | 71 |  | 0.67 (0.54, 0.84) | 0.76 (0.51, 1.15) |  | 1208 | 675 | 182 |  | 1.08 (0.84, 1.40) | 0.98 (0.68, 1.39) |
| 4 | 1406 | 495 | 52 |  | 0.68 (0.54, 0.86) | 0.44 (0.27, 0.69) |  | 2349 | 1170 | 343 |  | 0.84 (0.66, 1.09) | 0.83 (0.59, 1.17) |
| 5 | 749 | 179 | 13 |  | 0.41 (0.31, 0.54) | 0.19 (0.10, 0.38) |  | 1492 | 645 | 153 |  | 0.60 (0.46, 0.78) | 0.48 (0.33, 0.69) |
| P for trend |  |  |  |  | <0.001 | <0.001 |  |  |  |  |  | <0.001 | <0.001 |
| Every 1-point increment |  |  |  |  | 0.78 (0.72, 0.85) | 0.60 (0.50, 0.72) |  |  |  |  |  | 0.79 (0.73, 0.85) | 0.75 (0.68, 0.83) |

^*^ N: sample size of total subjects in each healthy lifestyle score group. Neither: subjects with neither hypertension nor T2DM, both: subjects with both hypertension and T2DM; either: subjects with only one disease of outcome.

^†^ OR (95% CI) was calculated by using multinominal logistic regression and with adjustment for age, sex, education, and marital status.

^‡^ *P*_-interaction_ (Either vs Neither) = 0.019, *P*_-interaction_ (Both vs Neither) = 0.001.

**Table S10 Association between healthy lifestyle score and the risk of hypertension and T2DM by using weighted lifestyle score**

| Healthy lifestyle score^§^ | N^*^ | |  | Effect | | |
| --- | --- | --- | --- | --- | --- | --- |
|  | Non-case group | Case group |  | Crude OR (95% CI) | Adjusted OR (95% CI) ^†^ | Adjusted OR (95% CI) ^‡^ |
| Hypertension |  |  |  |  |  |  |
| Tertile 1 (≤ 2.59) | 1197 | 1514 |  | 1.00 | 1.00 | 1.00 |
| Tertile 2 (2.59-4.47) | 2115 | 1986 |  | 0.74 (0.67, 0.82) | 0.67 (0.60, 0.75) | 0.68 (0.61, 0.76) |
| Tertile 3 (>4.47) | 1577 | 950 |  | 0.48 (0.43, 0.53) | 0.44 (0.39, 0.50) | 0.45 (0.40, 0.51) |
| *P* for trend |  |  |  | <0.001 | <0.001 | <0.001 |
| Every 1-point increment |  |  |  | 0.69 (0.65, 0.73) | 0.66 (0.62, 0.71) | 0.67 (0.63, 0.72) |
| T2DM |  |  |  |  |  |  |
| Tertile 1 (≤2.55) | 4214 | 933 |  | 1.00 | 1.00 | 1.00 |
| Tertile 2 (2.55-4.55) | 1094 | 164 |  | 0.68 (0.56, 0.81) | 0.62 (0.51, 0.75) | 0.67 (0.56, 0.81) |
| Tertile 3 (>4.55) | 2535 | 399 |  | 0.71 (0.63, 0.81) | 0.71 (0.62, 0.81) | 0.75 (0.66, 0.86) |
| *P* for trend |  |  |  | <0.001 | <0.001 | <0.001 |
| Every 1-point increment |  |  |  | 0.83 (0.78, 0.89) | 0.82 (0.77, 0.88) | 0.85 (0.80, 0.91) |

^*^ N represents sample size for non- case group or for case group.

^†^ Adjustment for age, sex, education, marital status, and retirement status.

^‡^ Additional adjustment for T2DM (for the association with hypertension) or hypertension (for the association with T2DM).

^§^ Weighted score = (β_1_ 🞨 factor_1_ + β_2_ 🞨 factor_3_ + β_3_ 🞨 factor_4_ + β_4_ 🞨 factor_4_ + β_5_ 🞨 factor _5_) 🞨(5 / sum of the β coefficients)

**Table S11 Sensitivity analyses for the association of healthy lifestyle score and the risk of comorbidity status of hypertension and T2DM using multinominal logistic regression**

| Healthy lifestyle score | N^*^ | | |  | Either vs Neither^†^ | |  | Both vs Neither^†^ | |
| --- | --- | --- | --- | --- | --- | --- | --- | --- | --- |
|  | Neither | Either | Both |  | Crude OR (95% CI) | Adjusted OR (95% CI) ^†^ |  | Crude OR (95% CI) | Adjusted OR (95% CI) ^†^ |
| By using median of LTPA in generating HLS | |  |  |  |  |  |  |  |  |
| 0-2 | 814 | 874 | 217 |  | 1.00 | 1.00 |  | 1.00 | 1.00 |
| 3 | 1166 | 1265 | 316 |  | 1.01 (0.89, 1.14) | 0.97 (0.84, 1.11) |  | 1.07 (0.84, 1.24) | 0.90 (0.72, 1.11) |
| 4 | 1514 | 1373 | 289 |  | 0.84 (0.75, 0.95) | 0.79 (0.68, 0.91) |  | 0.72 (0.59, 0.87) | 0.60 (0.48, 0.76) |
| 5 | 842 | 548 | 121 |  | 0.61 (0.52, 0.70) | 0.54 (0.46, 0.65) |  | 0.54 (0.42, 0.69) | 0.43 (0.32, 0.57) |
| *P* for trend |  |  |  |  | <0.001 | <0.001 |  | <0.001 | <0.001 |
| Every 1-point increment |  |  |  |  | 0.85 (0.82, 0.89) | 0.82 (0.78, 0.86) |  | 0.80 (0.75, 0.86) | 0.74 (0.68, 0.80) |
| By using BMI=25 kg/m2 as cut-off value in generating HLS | | |  |  |  |  |  |  |  |
| 0-2 | 373 | 477 | 114 |  | 1.00 | 1.00 |  | 1.00 | 1.00 |
| 3 | 834 | 928 | 244 |  | 0.87 (0.74, 1.03) | 0.81 (0.68, 0.97) |  | 0.96 (0.74, 1.23) | 0.81 (0.62, 1.07) |
| 4 | 1624 | 1598 | 353 |  | 0.77 (0.66, 0.90) | 0.68 (0.57, 0.81) |  | 0.71 (0.56, 0.90) | 0.55 (0.42, 0.72) |
| 5 | 1505 | 1057 | 232 |  | 0.55 (0.47, 0.64) | 0.48 (0.40, 0.58) |  | 0.50 (0.39, 0.65) | 0.38 (0.28, 0.51) |
| *P* for trend |  |  |  |  | <0.001 | <0.001 |  | <0.001 | <0.001 |
| Every 1-point increment |  |  |  |  | 0.81 (0.77, 0.85) | 0.78 (0.74, 0.82) |  | 0.77 (0.72, 0.83) | 0.71 (0.74, 0.82) |
| By excluding participants with BMI less than 18.5 kg/m^2^ | | |  |  |  |  |  |  |  |
| 0-2 | 397 | 514 | 127 |  | 1.00 | 1.00 |  | 1.00 | 1.00 |
| 3 | 884 | 1010 | 252 |  | 0.88 (0.75, 1.03) | 0.80 (0.67, 0.95) |  | 0.89 (0.70, 1.14) | 0.72 (0.55, 0.95) |
| 4 | 1563 | 1610 | 386 |  | 0.80 (0.69, 0.92) | 0.68 (0.57, 0.80) |  | 0.77 (0.61, 0.97) | 0.56 (0.43, 0.73) |
| 5 | 1251 | 824 | 166 |  | 0.51 (0.43, 0.60) | 0.42 (0.35, 0.51) |  | 0.41 (0.32, 0.54) | 0.28 (0.21, 0.39) |
| *P* for trend |  |  |  |  | <0.001 | <0.001 |  | <0.001 | <0.001 |
| Every 1-point increment |  |  |  |  | 0.80 (0.76, 0.83) | 0.75 (0.71, 0.79) |  | 0.75 (0.69, 0.81) | 0.70 (0.71, 0.79) |
| By excluding participants aged 75 years and above | |  |  |  |  |  |  |  |  |
| 0-2 | 416 | 475 | 112 |  | 1.00 | 1.00 |  | 1.00 | 1.00 |
| 3 | 926 | 860 | 211 |  | 0.81 (0.69, 0.96) | 0.79 (0.66, 0.94) |  | 0.85 (0.65, 1.09) | 0.77 (0.58, 1.02) |
| 4 | 1626 | 1427 | 303 |  | 0.77 (0.66, 0.89) | 0.71 (0.60, 0.85) |  | 0.69 (0.54, 0.88) | 0.57 (0.43, 0.76) |
| 5 | 1207 | 705 | 118 |  | 0.51 (0.44, 0.60) | 0.47 (0.39, 0.57) |  | 0.36 (0.27, 0.48) | 0.30 (0.21, 0.41) |
| *P* for trend |  |  |  |  | <0.001 | <0.001 |  | <0.001 | <0.001 |
| Every 1-point increment |  |  |  |  | 0.81 (0.77, 0.85) | 0.78 (0.74, 0.83) |  | 0.72 (0.67, 0.78) | 0.67 (0.60, 0.74) |
| By replacing the BMI with waist circumference | |  |  |  |  |  |  |  |  |
| 0-2 | 448 | 595 | 143 |  | 1.00 | 1.00 |  | 1.00 | 1.00 |
| 3 | 1035 | 1146 | 300 |  | 0.83 (0.72, 0.97) | 0.77 (0.65, 0.91) |  | 0.91 (0.72, 1.14) | 0.77 (0.60, 0.99) |
| 4 | 1776 | 1795 | 413 |  | 0.76 (0.66, 0.87) | 0.70 (0.59, 0.82) |  | 0.73 (0.59, 0.90) | 0.60 (0.47, 0.77) |
| 5 | 1077 | 524 | 87 |  | 0.36 (0.31, 0.43) | 0.37 (0.31, 0.45) |  | 0.25 (0.19, 0.34) | 0.24 (0.17, 0.34) |
| *P* for trend |  |  |  |  | <0.001 | <0.001 |  | <0.001 | <0.001 |
| Every 1-point increment |  |  |  |  | 0.74 (0.70, 0.77) | 0.74 (0.70, 0.78) |  | 0.67 (0.62, 0.72) | 0.65 (0.59, 0.71) |
| With additional adjustment for the number of dyslipidemia indicators | | | | | | | | | |
| 0-2 | 433 | 536 | 129 |  | 1.00 | 1.00 |  | 1.00 | 1.00 |
| 3 | 957 | 1035 | 253 |  | 0.87 (0.75, 1.02) | 0.83 (0.70, 0.98) |  | 0.82 (0.69, 0.97) | 0.79 (0.60, 1.03) |
| 4 | 1695 | 1665 | 395 |  | 0.79 (0.69, 0.92) | 0.73 (0.61, 0.86) |  | 0.71 (0.60, 0.84) | 0.65 (0.50, 0.84) |
| 5 | 1251 | 824 | 166 |  | 0.53 (0.46, 0.62) | 0.49 (0.41, 0.59) |  | 0.48 (0.40, 0.57) | 0.38 (0.28, 0.51) |
| *P* for trend |  |  |  |  | <0.001 | <0.001 |  | <0.001 | <0.001 |
| Every 1-point increment |  |  |  |  | 0.81 (0.78, 0.85) | 0.79 (0.75, 0.84) |  | 0.78 (0.74, 0.83) | 0.72 (0.66, 0.79) |

^*^ N: sample size of total subjects in each healthy lifestyle score group. BMI, body mass index; LTPA, leisure-time physical activity; HLS, healthy lifestyle score; Neither, subjects with neither hypertension nor T2DM; Both, subjects with both hypertension and T2DM; Either, subjects with only one disease of outcome.

^†^ Adjustment for age, sex, education, marital status, and retirement status

**Table S12 Association between healthy lifestyle score and the risk of hypertension and T2DM by using median of LTPA in generating HLS**

| Healthy lifestyle score | N^*^ | |  | Effect | | |
| --- | --- | --- | --- | --- | --- | --- |
|  | Non-case group | Case group |  | Crude OR (95% CI) | Adjusted OR (95% CI) ^†^ | Adjusted OR (95% CI) ^‡^ |
| Hypertension |  |  |  |  |  |  |
| 0-2 | 905 | 1000 |  | 1.00 | 1.00 | 1.00 |
| 3 | 1333 | 1414 |  | 0.96 (0.85, 1.08) | 0.90 (0.79, 1.03) | 0.90 (0.79, 1.03) |
| 4 | 1727 | 1449 |  | 0.76 (0.68, 0.85) | 0.70 (0.61, 0.80) | 0.70 (0.61, 0.81) |
| 5 | 924 | 587 |  | 0.57 (0.50, 0.66) | 0.52 (0.44, 0.61) | 0.53 (0.45, 0.63) |
| *P* for trend |  |  |  | <0.001 | <0.001 | <0.001 |
| Every 1-point increment |  |  |  | 0.83 (0.80, 0.87) | 0.80 (0.76, 0.84) | 0.81 (0.77, 0.85) |
| T2DM |  |  |  |  |  |  |
| 0-2 | 1597 | 308 |  | 1.00 | 1.00 | 1.00 |
| 3 | 2264 | 483 |  | 1.11 (0.95, 1.29) | 1.02 (0.87, 1.21) | 1.03 (0.87, 1.22) |
| 4 | 2674 | 502 |  | 0.97 (0.83, 1.14) | 0.88 (0.74, 1.04) | 0.91 (0.76, 1.08) |
| 5 | 1308 | 203 |  | 0.80 (0.66, 0.97) | 0.70 (0.56, 0.86) | 0.74 (0.60, 0.92) |
| *P* for trend |  |  |  | 0.013 | <0.001 | 0.003 |
| Every 1-point increment |  |  |  | 0.93 (0.88, 0.98) | 0.89 (0.83, 0.94) | 0.91 (0.85, 0.97) |

^*^ N represents sample size for non- case group or for case group.

^†^ Adjustment for age, sex, education, marital status, and retirement status.

^‡^ Additional adjustment for T2DM (for the association with hypertension) or hypertension (for the association with T2DM).

**Table S13 Association between healthy lifestyle score and the risk of hypertension and T2DM by using BMI=25 kg/m2 as cut-off value in generating HLS**

| Healthy lifestyle score | N^*^ | |  | Effect | | |
| --- | --- | --- | --- | --- | --- | --- |
|  | Non-case group | Case group |  | Crude OR (95% CI) | Adjusted OR (95% CI) ^†^ | Adjusted OR (95% CI) ^‡^ |
| Hypertension |  |  |  |  |  |  |
| 0-2 | 425 | 539 |  | 1.00 | 1.00 | 1.00 |
| 3 | 937 | 1069 |  | 0.90 (0.77, 1.05) | 0.83 (0.70, 0.99) | 0.84 (0.70, 0.99) |
| 4 | 1856 | 1719 |  | 0.73 (0.63, 0.84) | 0.64 (0.54, 0.75) | 0.64 (0.54, 0.76) |
| 5 | 1671 | 1123 |  | 0.53 (0.46, 0.61) | 0.47 (0.39, 0.56) | 0.48 (0.40, 0.57) |
| *P* for trend |  |  |  | <0.001 | <0.001 | <0.001 |
| Every 1-point increment |  |  |  | 0.80 (0.76, 0.83) | 0.76 (0.72, 0.81) | 0.77 (0.73, 0.81) |
| T2DM |  |  |  |  |  |  |
| 0-2 | 798 | 166 |  | 1.00 | 1.00 | 1.00 |
| 3 | 1659 | 347 |  | 1.01 (0.82, 1.23) | 0.92 (0.74, 1.13) | 0.93 (0.75, 1.15) |
| 4 | 2990 | 585 |  | 0.94 (0.78, 1.14) | 0.82 (0.67, 1.02) | 0.86 (0.70, 1.06) |
| 5 | 2396 | 398 |  | 0.80 (0.66, 0.98) | 0.68 (0.54, 0.85) | 0.73 (0.59, 0.92) |
| *P* for trend |  |  |  | 0.004 | <0.001 | 0.002 |
| Every 1-point increment |  |  |  | 0.92 (0.87, 0.97) | 0.88 (0.82, 0.94) | 0.90 (0.84, 0.96) |

^*^ N represents sample size for non- case group or for case group.

^†^ Adjustment for age, sex, education, marital status, and retirement status.

^‡^ Additional adjustment for T2DM (for the association with hypertension) or hypertension (for the association with T2DM).

**Table S14Association between healthy lifestyle score and the risk of hypertension and T2DM after replacing the BMI with waist** **circumference**

| Healthy lifestyle score^§^ | N^*^ | |  | Effect | | |
| --- | --- | --- | --- | --- | --- | --- |
|  | Non-case group | Case group |  | Crude OR (95% CI) | Adjusted OR (95% CI) ^†^ | Adjusted OR (95% CI) ^‡^ |
| Hypertension |  |  |  |  |  |  |
| 0-2 | 404 | 563 |  | 1.00 | 1.00 | 1.00 |
| 3 | 939 | 1122 |  | 0.86 (0.73, 1.00) | 0.78 (0.65, 0.92) | 0.78 (0.66, 0.93) |
| 4 | 1849 | 1787 |  | 0.69 (0.60, 0.80) | 0.63 (0.53, 0.74) | 0.64 (0.54, 0.75) |
| 5 | 1697 | 978 |  | 0.41 (0.36, 0.48) | 0.42 (0.35, 0.51) | 0.43 (0.36, 0.52) |
| *P* for trend |  |  |  | <0.001 | <0.001 | <0.001 |
| Every 1-point increment |  |  |  | 0.73 (0.70, 0.77) | 0.75 (0.71, 0.79) | 0.76 (0.72, 0.80) |
| T2DM |  |  |  |  |  |  |
| 0-2 | 789 | 178 |  | 1.00 | 1.00 | 1.00 |
| 3 | 1693 | 368 |  | 0.96 (0.79, 1.18) | 0.87 (0.71, 1.08) | 0.89 (0.73, 1.10) |
| 4 | 3031 | 605 |  | 0.88 (0.74, 1.07) | 0.78 (0.64, 0.95) | 0.81 (0.66, 1.00) |
| 5 | 2330 | 345 |  | 0.66 (0.54, 0.80) | 0.59 (0.47, 0.74) | 0.64 (0.51, 0.81) |
| *P* for trend |  |  |  | <0.001 | <0.001 | <0.001 |
| Every 1-point increment |  |  |  | 0.86 (0.82, 0.92) | 0.84 (0.79, 0.90) | 0.86 (0.81, 0.92) |

^*^ N represents sample size for non- case group or for case group.

^†^ Adjustment for age, sex, education, marital status, and retirement status.

^‡^ Additional adjustment for T2DM (for the association with hypertension) or hypertension (for the association with T2DM).

^§^ Replaced BMI with waist circumference (WC), the WC healthy group was < 90 cm (men) or 85 cm (women).

**Table S15 Association between healthy lifestyle score and the risk of hypertension and T2DM after excluding participants with BMI less than 18.5 kg/m^2^**

| Healthy lifestyle score | N^*^ | |  | Effect | | |
| --- | --- | --- | --- | --- | --- | --- |
|  | Non-case group | Case group |  | Crude OR (95% CI) | Adjusted OR (95% CI) ^†^ | Adjusted OR (95% CI) ^‡^ |
| Hypertension |  |  |  |  |  |  |
| 0-2 | 453 | 585 |  | 1.00 | 1.00 | 1.00 |
| 3 | 995 | 1151 |  | 0.90 (0.77, 1.04) | 0.80 (0.68, 0.95) | 0.81 (0.68, 0.96) |
| 4 | 1795 | 1764 |  | 0.76 (0.66, 0.87) | 0.63 (0.53, 0.75) | 0.64 (0.54, 0.76) |
| 5 | 1382 | 859 |  | 0.48 (0.41, 0.56) | 0.40 (0.33, 0.48) | 0.41 (0.34, 0.49) |
| *P* for trend |  |  |  | <0.001 | <0.001 | <0.001 |
| Every 1-point increment |  |  |  | 0.78 (0.75, 0.81) | 0.73 (0.69, 0.77) | 0.74 (0.70, 0.78) |
| T2DM |  |  |  |  |  |  |
| 0-2 | 855 | 183 |  | 1.00 | 1.00 | 1.00 |
| 3 | 1783 | 363 |  | 0.95 (0.78, 1.16) | 0.86 (0.70, 1.06) | 0.87 (0.71, 1.08) |
| 4 | 2941 | 618 |  | 0.98 (0.82, 1.18) | 0.84 (0.69, 1.03) | 0.88 (0.72, 1.08) |
| 5 | 1944 | 297 |  | 0.71 (0.58, 0.87) | 0.59 (0.47, 0.75) | 0.65 (0.52, 0.82) |
| *P* for trend |  |  |  | <0.001 | <0.001 | <0.001 |
| Every 1-point increment |  |  |  | 0.91 (0.85, 0.96) | 0.85 (0.80, 0.91) | 0.88 (0.82, 0.94) |

^*^ N represents sample size for non- case group or for case group.

^†^ Adjustment for age, sex, education, marital status, and retirement status.

^‡^ Additional adjustment for T2DM (for the association with hypertension) or hypertension (for the association with T2DM).

**Table S16 Association between healthy lifestyle score and the risk of hypertension and T2DM after excluding participants aged 75 years and above**

| Healthy lifestyle score | N^*^ | |  | Effect | | |
| --- | --- | --- | --- | --- | --- | --- |
|  | Non-case group | Case group |  | Crude OR (95% CI) | Adjusted OR (95% CI) ^†^ | Adjusted OR (95% CI) ^‡^ |
| Hypertension |  |  |  |  |  |  |
| 0-2 | 474 | 529 |  | 1.00 | 1.00 | 1.00 |
| 3 | 1031 | 966 |  | 0.84 (0.72, 0.98) | 0.81 (0.69, 0.96) | 0.75 (0.61, 0.91) |
| 4 | 1859 | 1497 |  | 0.72 (0.63, 0.83) | 0.66 (0.56, 0.79) | 0.63 (0.51, 0.77) |
| 5 | 1332 | 698 |  | 0.47 (0.40, 0.55) | 0.44 (0.36, 0.53) | 0.39 (0.30, 0.49) |
| *P* for trend |  |  |  | <0.001 | <0.001 | <0.001 |
| Every 1-point increment |  |  |  | 0.78 (0.75, 0.82) | 0.76 (0.72, 0.80) | 0.74 (0.69, 0.80) |
| T2DM |  |  |  |  |  |  |
| 0-2 | 833 | 170 |  | 1.00 | 1.00 | 1.00 |
| 3 | 1681 | 316 |  | 0.92 (0.75, 1.13) | 0.87 (0.70, 1.07) | 0.82 (0.69, 0.97) |
| 4 | 2820 | 536 |  | 0.93 (0.77, 1.13) | 0.83 (0.67, 1.03) | 0.67 (0.57, 0.79) |
| 5 | 1787 | 243 |  | 0.67 (0.54, 0.82) | 0.58 (0.46, 0.75) | 0.45 (0.37, 0.55) |
| *P* for trend |  |  |  | <0.001 | <0.001 | <0.001 |
| Every 1-point increment |  |  |  | 0.89 (0.84, 0.95) | 0.85 (0.79, 0.91) | 0.77 (0.72, 0.81) |

^*^ N represents sample size for non- case group or for case group.

^†^ Adjustment for age, sex, education, marital status, and retirement status.

^‡^ Additional adjustment for T2DM (for the association with hypertension) or hypertension (for the association with T2DM).

**Table S17 Association between healthy lifestyle score and the risk of hypertension and T2DM with additional adjustment for the number of dyslipidemia indicators**

| Healthy lifestyle score | N^*^ | |  | OR (95% CI) | | |
| --- | --- | --- | --- | --- | --- | --- |
|  | Non-case group | Case group |  | Crude OR | Adjusted OR^†^ | Adjusted OR ^‡^ |
| Hypertension |  |  |  |  |  |  |
| 0-2 | 496 | 602 |  | 1.00 | 1.00 | 1.00 |
| 3 | 1070 | 1175 |  | 0.90 (0.78, 1.05) | 0.86 (0.73, 1.01) | 0.87 (0.73, 1.02) |
| 4 | 1941 | 1814 |  | 0.77 (0.67, 0.88) | 0.69 (0.59, 0.81) | 0.70 (0.59, 0.83) |
| 5 | 1382 | 859 |  | 0.51 (0.44, 0.59) | 0.48 (0.40, 0.57) | 0.49 (0.41, 0.59) |
| *P* for trend |  |  |  | <0.001 | <0.001 | <0.001 |
| Every 1-point increment |  |  |  | 0.80 (0.76, 0.83) | 0.77 (0.73, 0.82) | 0.78 (0.74, 0.82) |
| T2DM |  |  |  |  |  |  |
| 0-2 | 906 | 192 |  | 1.00 | 1.00 | 1.00 |
| 3 | 1879 | 366 |  | 0.92 (0.76, 1.11) | 0.85 (0.70, 1.05) | 0.87 (0.71, 1.07) |
| 4 | 3114 | 641 |  | 0.97 (0.81, 1.16) | 0.88 (0.73, 1.08) | 0.91 (0.74, 1.11) |
| 5 | 1944 | 297 |  | 0.72 (0.59, 0.88) | 0.67 (0.54, 0.84) | 0.71 (0.56, 0.89) |
| *P* for trend |  |  |  | 0.003 | 0.001 | 0.006 |
| Every 1-point increment |  |  |  | 0.92 (0.86, 0.97) | 0.89 (0.84, 0.96) | 0.91 (0.85, 0.97) |

^*^ N represents sample size for non-case group or for case group.

^†^ Adjustment for age, sex, education, marital status, and retirement status, T2DM (for the association with hypertension) or hypertension (for the association with T2DM).

^‡^ Additional adjustment for the number of dyslipidemia indicators.
